# Do professional perspectives on evidence-based smoking cessation methods align? A Delphi study among researchers and healthcare professionals

**DOI:** 10.1093/her/cyab022

**Published:** 2022-01-22

**Authors:** Daniëlle N Zijlstra, Ciska Hoving, Catherine Bolman, Jean W M Muris, Hein De Vries

**Affiliations:** Department of Health Promotion, CAPHRI, Maastricht University, Peter Debyeplein 1, Maastricht 6229 HA, Netherlands; Department of Health Promotion, CAPHRI, Maastricht University, Peter Debyeplein 1, Maastricht 6229 HA, Netherlands; Department of Psychology, Open University of the Netherlands, P.O. Box 2960, Heerlen 6401 DL, Netherlands; Department of General Practice, CAPHRI, Maastricht University, Peter Debyeplein 1, Maastricht 6229 HA, Netherlands; Department of Health Promotion, CAPHRI, Maastricht University, Peter Debyeplein 1, Maastricht 6229 HA, Netherlands

## Abstract

The use of evidence-based smoking cessation interventions (SCIs) can significantly increase the number of successful smoking cessation attempts. To obtain an overview of the knowledge and viewpoints on the effectiveness and use of SCIs, a three-round online Delphi study was conducted among researchers and primary care professionals (PCPs). The four objectives of this study are to gain an overview of (i) the criteria important for recommending SCIs, (ii) the perceptions of both groups on the effectiveness of SCIs, (iii) the factors to consider when counseling different (high-risk) groups of smokers and (iv) the perceptions of both groups on the use of e-cigarettes as an SCI. We found a high level of agreement within groups on which smoker characteristics should be considered when recommending an SCI to smokers. We also found that PCPs display a lower degree of consensus on the effectiveness of SCIs. Both groups see a value in the use of special protocols for different (high-risk) groups of patients, but the two groups did not reach consensus on the use of e-cigarettes as a means to quit. Making an inventory of PCPs’ needs regarding SCIs and their usage may provide insight into how to facilitate a better uptake in the primary care setting.

## Background

Globally, smoking continues to be a leading cause of preventable morbidity and premature death [1]. Although in the Netherlands the prevalence of smoking has decreased, 21.7% of adults (3.0 million people) still smoked in 2019 [2]. Each year, around one-third of Dutch smokers report making at least one serious (at least 24 h of no smoking) quit attempt [2]. However, only a small percentage of smokers manages to quit long term [3, 4].

Each year, around two-thirds of Dutch smokers visit their primary care practice [5]. As such, Dutch primary care professionals (PCPs) are well-positioned to initiate smoking cessation. In practice, the provision of smoking cessation support in Dutch primary care practices has to a large extent shifted from general practitioners (GPs) to trained practice nurses (PNs) [6, 7], who counsel and treat patients on an independent basis but operate under the responsibility of a GP [8] and rely to a large extent on the use of evidence-based guidelines and protocols [8].

In the Netherlands, the Dutch College of General Practitioners (Nederlands Huisartsen Genootschap) is responsible for developing national smoking cessation guidelines for the primary care setting [9], similar to for example the ‘5 As’(ask, advise, assess, assist and arrange) [10].

Although a majority of PNs report using evidence-based guidelines to structure their smoking cessation counseling (SCC) [6], important counseling elements such as increasing patients’ motivation and removing cessation barriers are not always implemented [5, 6, 11, 12]. PNs list several psychological (e.g. low self-efficacy to increase patient motivation) and practical barriers (e.g. difficulties in providing patients with relevant and up-to-date information) which prevent them from (fully) adhering to the guidelines [6]. Another study found several barriers, such as SCC being too time-consuming and insufficient reimbursements for treating smoking patients without smoking-related illnesses [11]. Similar and other barriers have been found in the primary care settings in other countries [13, 14].

These guidelines can be used by PCPs to structure consultations with smoking patients and discuss a range of smoking cessation interventions (SCIs), focusing not only on behavioral counseling by PCPs (e.g. face-to-face counseling) but also discussing behavioral counseling outside of the primary care setting (e.g. eHealth or counseling in groups) and pharmacological interventions (e.g. nicotine replacement therapy; NTR) [15]. Although a wide range of SCIs with a strong evidence base is available [16], these interventions often remain underused [17]. The use of evidence-based interventions to support smoking cessation can significantly increase the success rate of quit attempts [15].

This study was conducted as part of a needs assessment in the development of a referral aid which aims to support PCPs in their referral of smoking patients to SCIs. To this end, we were interested in exploring the knowledge and viewpoints on the effectiveness and use of SCIs among PCPs., To our knowledge, no prior research has been conducted on these topics. Therefore, the first objective of this study is to gain an overview of criteria (both patient and intervention related) that are perceived to be important to consider when recommending an SCI to individual smoking patients. The second objective is to gain an overview of perceptions on the effectiveness of existing SCIs. As smokers who visit PCPs often display smoking-related complaints such as asthma or chronic obstructive pulmonary disease, the third objective is to gain an overview of criteria that are important to consider when counseling different (high-risk) groups of smoking patients. Other high-risk groups of smokers, as also indicated in the Dutch guidelines for smoking cessation, are pregnant smokers, smokers with a low social economic status (low-SES) and smokers with a low motivation to quit. The fourth and last objective pertains to the use of e-cigarettes. As e-cigarettes have not been proven to be an effective and safe smoking cessation aid, their use as an SCI is discouraged by the Dutch smoking cessation guidelines. However, to our knowledge, no research has been done on PCPs adherence to this recommendation, and therefore, our last objective is to gain an overview of perceptions on the use of e-cigarettes as a means to quit.

As smoking cessation is a complex health issue which is not only tackled by the primary care setting, we decided to include not only PCPs but also researchers from the field of tobacco control. Other similar structured studies have suggested that researchers are sometimes more able to identify overarching themes and bring a unique perspective [18, 19]. Studying the perceptions of researchers and PCPs simultaneously may give a unique insight into the challenges of the current daily practice and provide solutions to eliminate certain barriers, while also allowing us to pinpoint potential evidence-practice gaps [20]. For this purpose, we used a three-round Delphi study to identify topics that are related to SCIs and on which those consensus does or does not exist among two different sets of experts.

## Methods

### Study design

The Delphi method is a technique for structuring communication in order to derive consensus on certain subjects for which scientific evidence is limited or conflicting by involving a panel of knowledgeable experts or individuals [21, 22]. The three rounds of this Delphi study were conducted in the period from October 2017 to April 2018. Participants who completed both the second and third rounds were awarded a €20 gift voucher.

#### First round

Through a database search in PsycINFO, PubMed and Google Scholar, 63 researchers (national and international) were identified who had (co-)authored at least five papers on a topic related to smoking cessation in the field of health promotion, behavior change and/or addiction in the previous five years (convenience sampling).

The Dutch Healthcare Chart (www.zorgkaartnederland.nl), a review site of Dutch care providers and care facilities, was used to identify 21 PCPs (both GPs and PNs) who were employed in a primary care practice at the time of the study and who regularly (i.e. at least once a week) offered smoking cessation advice and counseling (i.e. actively assisting patients in a quit attempt keeping with the Dutch guidelines for smoking cessation). Only providing a brief cessation advice was used as an exclusion criterion. The questionnaires for the researchers were formulated in English, and the questionnaires for the PCPs were formulated in Dutch (in all three rounds). The questionnaires were otherwise identical.

The first-round questionnaire consisted of 15 open-ended questions covering five main topics (in accordance with the study’s objectives). Participants were each asked to (i) list patient characteristics that should be taken into account when recommending an SCI to an individual patient (patient characteristics), (ii) list criteria that should be met by an SCI when recommending an SCI to an individual patient in order for it to be most effective (intervention characteristics), (iii) list existing SCIs they perceive to be effective, (iv) list factors that should be taken into account when counseling different (high-risk) groups of smoking patients and (v) to voice their opinion on the use of e-cigarettes as a means to quit.

The responses to the open-ended questions were qualitatively analyzed by two researchers, using the Framework method [23] to merge individual responses into closed-ended statements and questions that were used as input for the second-round questionnaire. Duplicate items were deleted, and semantically similar items were merged. Inter-rater correlation was then calculated using Cohen’s Kappa (K). This resulted in an intercoder reliability of 99% (percentage of agreement) and a K = 0.71, indicating a substantial level of agreement between both researchers [24].

#### Second round

All participants who had completed the first round were invited to participate in the second round. An additional 215 researchers were identified using the same strategy as used in the first round. An additional 174 PCPs were identified through the ‘Dutch Register for Qualified Smoking Cessation Professionals’ (Kwaliteitsregister Stoppen met Roken) (DRQSCP) [25]. All 409 potential participants were then invited via email to participate in the second and third rounds.

The second-round questionnaire consisted of 63 closed-ended statements (see Supplementary Appendix—Table SII), which were based on the responses to the open-ended questions from the first round. For all questions and statements, answers were given on a 7-point Likert Scale (depending on the type of item 1 = strongly disagree to 7 = strongly agree, 1 = not important at all to 7 = very important, or 1 = not effective at all to 7 = very effective).

For each of the 63 items, each group’s level (depending on the type of item) of agreement was analyzed by calculating the median score (Mdn), and each group’s level of consensus was analyzed by calculating the interquartile range (IQR). A cut-off point of an Mdn of ≥ 6 was used. The IQR represents the level of consensus by calculating the difference between the 25th and 75th percentiles, with a smaller value indicating a smaller data spread and a higher level of consensus. An IQR ≤1 is considered to be indicative of good consensus on a 7-point scale [26].

#### Third round

All 78 participants who had completed the second round were invited to participate in the third round, using the same procedure as used in the second round.

The third-round questionnaire consisted of the items from the second round on which consensus had not yet been reached (IQR ≥1) in the second round. For each item, the group median and IQR from the second round was presented to the participants.

For each item, the Mdn and IQR were calculated. The between-group consensus was analyzed using Wilcoxon signed-rank sum tests, as the data were not normally distributed.

## Results

**Fig. 1.**
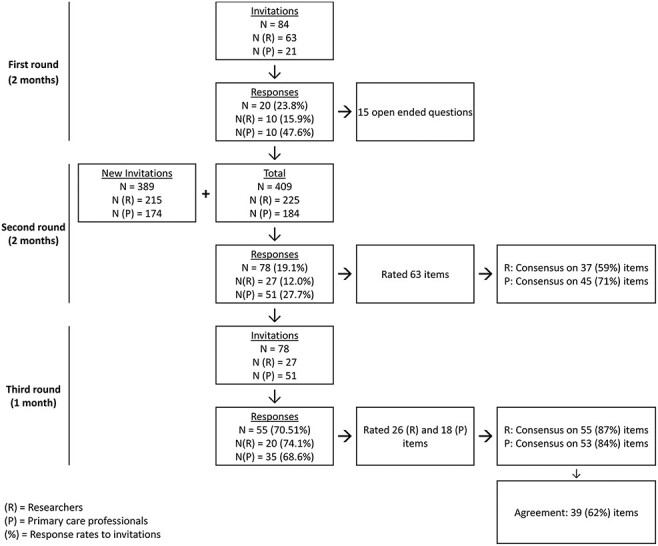
Overview of the recruitment process.

Of the 84 potential participants who were approached, 20 completed the first round (24% response rate); 10 in each group (see Fig. 1 for an overview of the recruitment process and the response rates). In the first round, the PCPs group consisted solely of PNs, who provided on average 8.2 h of smoking cessation advice and counseling each month. The researchers group varied in experience level, ranging from junior researchers to full professors.

In the first round, (see Table I for all Characteristics of the participants) the participants identified 12 patient characteristics (e.g. the patient’s preference for an SCI) and 13 intervention characteristics (e.g. the intervention continues over a longer period of time) which they deemed important when recommending an SCI to individual patients. They also listed 13 different SCIs and 22 factors that should be taken into account when counseling four different (high-risk) groups of patients. Lastly, three different statements could be derived from participants’ opinions on the use of e-cigarettes as a means to quit (e.g. e-cigarettes can be recommended as a SCI but not as the most preferred option).

**Table I.** Characteristics of the participants

	Round 1	Round 2	Round 3
Current occupation of health professionals			
GP (%)	0 (0.0)	23 (45.1)	14 (40.0)
PN (%)	10 (100.0)	28 (54.9)	21 (60.0)
Mean monthly hours of providing smoking cessation advice (SD)	8.2 (3.1)	5.6 (8.2)	4.7 (4.5)
Current occupation of researchers			
Junior researchers (%)	6 (60.0)	13 (48.1)	6 (30.0)
Assistant professor (%)	3 (30.0)	4 (14.8)	5 (25.0)
Associate professor (%)	0 (0.0)	6 (22.2)	4 (20.0)
Full professor (%)	1 (10.0)	4 (14.8)	5 (25.0)
Field of expertise of researchers^a^			
Smoking behavior and tobacco use	3	21 (77.8)	16 (79.2)
(Development of) Tobacco interventions	7	14 (51.9)	13 (61.9)
(Development of) Health promotion interventions	3	11 (40.7)	11 (52.4)
Other, but relevant for this study^b^	1	5 (18.5)	0 (0.0)

a Researchers were able to mark more than one field of expertise.

b For example, reaching low socioeconomic status groups, lung cancer screening trials, development of tobacco control policy and guideline development.

These 63 items identified in the first round were converted into closed-ended statements and statements and included in the second-round questionnaire. In the second round, 27 researchers and 51 PCPs (both GPs and PNs) participated. The researchers group then reached consensus (IQR ≤ 1) on 37 (59%) items and the PCPs group reached consensus on 45 (71%) items (all items are listed in Supplementary Appendix—Table SII).

In the third round, 20 researchers and 35 PCPs (GPs and PNs) participated. Consensus was then reached by the researchers’ group on 55 (87%) items and by the PCPs group on 53 (84%) items. Finally, between-group consensus (sig ≥ 0.05) was reached on 39 (62%) items. The results of the Wilcoxon signed-rank sum tests can be found in Supplementary appendix—Table SIII. Detailed results per topic are discussed below.

### Patient characteristics that should be taken into account when choosing recommending an SCI (patient characteristics)

The researchers group reached consensus (IQR ≤ 1) on the level of importance of all 12 listed characteristics in the second round. Of the 12 characteristics, six were rated as important or very important (Mdn ≥ 6) (see Supplementary appendix—Table SII). Over the course of rounds two and three, the PCPs group also reached consensus on the level of importance of all 12 items. With the exception of the patient’s level of nicotine addiction, the PCPs group rated the same items as important or very important as the researchers group. Between-group consensus was reached on eight items (67% consensus), with three characteristics regarding the smoker’s preference, experience with previous attempts and motivation rated as very important (Mdn ≥ 6) (see Supplementary Appendix—Table SIII).

### Criteria that should be met by an SCI when choosing recommending an SCI for to an individual patient (intervention characteristics)

The researchers group reached consensus on the level of importance of 12 of the 13 listed criteria over the course of rounds two and three; no consensus was reached on the inclusion of highly detailed information on smoking cessation in the intervention. Seven criteria were rated as important or very important (Mdn ≥ 6). The PCPs group also reached consensus on the level of importance of 12 of the 13 items over the course of rounds two and three (all except the item regarding the length of the intervention). Four items were rated as important or very important. Between-group consensus was reached on nine items (69%) in which high motivation scored as the most important item (Mdn = 6.5) and the intervention including fewer session as the least important (Mdn = 4.5).

### How is the effectiveness of existing SCIs perceived?

The researchers group reached consensus on the level of effectiveness of 10 of the 13 listed SCIs over the course of rounds two and three. Four of these were rated as effective or very effective (pharmacotherapy, NRT, brief cessation advice by a healthcare professional without additional interventions, and counseling in groups) (Mdn ≥ 6).

The PCPs group reached consensus on the level of effectiveness of eight of the thirteen items over the course of rounds two and three. Only two items were rated as effective or very effective: (i) pharmacotherapy and (ii) brief cessation advice by a health-care professional without additional interventions. Combining the results on importance and consensus, we can conclude that consensus was only reached on the latter. Between-group consensus was reached on seven out of thirteen interventions, with pharmacotherapy scoring the highest on effectiveness (Mdn = 6) and e-cigarettes the lowest (Mdn ≤ 3).

### Factors that should be taken into account when counseling different (high-risk) groups of smoking patients

Participants were asked to rate the level of importance of 22 factors divided over four (high-risk) groups of smoking patients.

#### Patients with smoking-related complaints

The researchers group reached consensus on the level of importance of five of the six listed factors over the course of rounds two and three and rated these factors as important or very important (Mdn ≥ 6). The PCPs group reached consensus on the level of importance of all six items in round two and rated the same five items as important or very important as the researchers. Between-group consensus was reached on four items with the most important factors being that the smoker should be informed about his or her health risk (both Mdn = 6.5) (67% consensus).

#### Pregnant patients

Both groups reached consensus on the importance of five of the six listed factors (consensus was reached in round two); neither group reached consensus on providing pregnant patients with the same cessation support as non-pregnant patients. Except for this factor, both groups rated the other five factors as important or very important (Mdn ≥ 6). Between-group consensus was reached on two items with the most important factor being that the smoker should be informed of the risks of smoking during pregnancy (Mdn ≥ 6.5) (33% consensus).

#### Patients with a low SES

The researchers group reached consensus on the level of importance of three of the five listed factors over the course of rounds two and three. These factors were also rated as important or very important (Mdn ≥ 6). The PCPs group reached consensus on the level of importance of four of the five items over the course of rounds two and three and also rated these as important. Between-group consensus was reached on all items, with ‘the smoker should be informed about his or her health risk’ scoring the highest (Mdn ≥ 6.5) (100% consensus).

#### Patients with a low motivation to quit

Both groups reached consensus on the level of importance of four of the five listed factors (consensus was reached in round two) and rated those factors as important or very important (Mdn ≥ 6). Between-group consensus was reached on four items, with the item on focusing the counseling on increasing motivation scoring the highest (Mdn ≥ 6.0) (80% consensus).

### The use of e-cigarettes as a means to quit

The researchers group reached consensus on their level of agreement on only one of the three statements on the use of e-cigarettes as a means to quit (consensus was reached in round two): informing patients fully about the risks of e-cigarettes before talking about them as an SCI. This statement was also the only one on which agreement with the statement was reached. The group of PCPs did not reach consensus on the statements. Between-group consensus was reached on one statement, namely, recommending e-cigarettes as a means to quit but not as the most preferred option.

## Discussion

### Main findings

The four objectives of this study were to gain an overview of (i) the criteria important for recommending SCIs, (ii) the perceptions on the effectiveness of SCIs, (iii) the criteria important for counseling different (high-risk) groups of smokers and (iv) the perceptions on the use of e-cigarettes. These topics will be discussed below.

First, consensus within both groups was exceptionally high on the level of importance of the different criteria for recommending an SCI to individual patients. Other studies have also found that the socio-economic status and smokers’ experience with previous cessation attempts play a significant role in successful smoking cessation [27, 28]. It is also known that raising the smokers motivation to quit, e.g. through the use of motivational interviewing techniques, during SCC can facilitate successful smoking cessation [29, 30]. The Dutch guidelines [9] also advise PCPs to discuss previous quit attempts and to inquire after the reasons why these failed in order to adapt treatment accordingly.

Second, the PCPs group only reached consensus on the level of effectiveness of one SCI: a brief cessation advice by a healthcare professional without additional interventions. A brief quit advice by a health-care professional has been shown to significantly increase smoking abstinence, regardless of the patient’s level of motivation [31]. This corresponds with the first step of the Dutch smoking cessation guidelines. However, the combination of two or more evidence-based SCIs increases the chance of a successful quit attempt [32, 33]. The use of multiple SCIs can help to increase smoking abstinence while also providing a wider range of options for smokers who are not able to visit their primary care practice or who have other preferences for SCC. The use of multiple SCIs can also take the form of in-practice counseling combined with an out-practice intervention such as eHealth or supplemental NRT.

Neither group rated the five non-evidence-based interventions (e-cigarettes, acupuncture, laser therapy, relaxation exercises and quitting without any form of cessation support) to be effective. Noted should be, however, that the level of effectiveness of three of these SCIs (i.e. acupuncture, laser therapy, and quitting without any form of cessation support) was rated higher in the PCPs group than in the researcher’s group. A possible explanation is that PCPs’ vision on the effectiveness is based on positive (personal) experiences. Further studies should investigate whether this is the case, how this vision can be adjusted and how to best discuss non-evidence-based interventions with patients.

Third, we can conclude that both groups see some value in providing additional cessation support to the four (high-risk) groups of patients. As smoking prevalence is higher among disadvantaged groups and disadvantaged smokers may face higher exposure to tobacco’s harms, they might have different needs related to cessation support [34–36] and the necessity of providing additional support to pregnant patients is widely recognized as smoking is associated with risks for both the patient and the unborn child [37]. Both groups indicated that smokers not motivated to quit should also be targeted, with the PCPs group stating that they find it important to increase motivation and use motivational interview techniques [29, 30].

Lastly, concerning the use of e-cigarettes, almost no consensus within or between the groups was reached. Possible explanations may be that research is still inconclusive on the use of e-cigarettes as a means to quit [38] that the quality and composition of e-cigarettes is highly variable [39] and that no concrete long-term effects in terms of effectiveness, safety and addiction are known [38, 40, 41].

### Practice implications

First, with regard to high-risk groups of smokers, our results indicated that both groups found it important to provide additional support to high-risk groups of patients. Referring high-risk groups, such as older smokers [42] or patients with multimorbidity diseases [43], to extended cognitive behavioral therapy increases the chances of a successful quit attempt, so this additional support is very valuable. Yet, in practice, PCPs do not always manage to pay extra attention to these group because of a lack of self-efficacy or time [44, 45] or a lower motivation among these type of patients [46–48]. As stated earlier, motivational interview techniques [29, 30] can help PCPs to increase motivation as well as convince smokers of their increased health risk (especially during pregnancy). Although the Dutch guidelines for smoking cessation mention low motivation, smoking-related diseases and low-SES, specific support directions are only provided for pregnant smokers. By adding support directions for the other high-risk groups, including information on the use of SCIs among high-risk groups (e.g. information on interventions designed for smokers with low language skills or interventions specifically targeted to pregnant woman), PCPs will be better able to support these groups, which may lead to higher quit percentages within these groups.

Second, while researchers reached consensus on the effectiveness on 10 out of 13 listed SCIs, PCPs only reached consensus on eight. In addition, as mentioned before, PCPs tend to rate the effectiveness of several non-evidence-based SCIs higher than the researchers’ group, which may indicate an evidence-practice gap. An evidence-practice gap sometimes is associated with a lack of knowledge or formal training in CSS [49]. Despite the fact that most Dutch PCPs are registered in the DRQSCP [25], this does not necessarily prevents misconceptions about the effectiveness of SCIs and this may imply the need for training programs or supplemental materials in order to fully acquaint PCPs with SCIs. To our knowledge, no prior research has been conducted on the perceptions of the effectiveness of SCIs among GPs and other PCPs.

Lastly, communication with PCPs on recent scientific findings may also reduce the uncertainty surrounding cigarettes, breaching the evidence-practice gap [20, 50]. While the Dutch national smoking cessation guidelines advise against the use of e-cigarettes [51], they do not elaborate on how to approach patients who express a desire to use them as an SCI. Until a clear outcome on the use of e-cigarettes as an SCI is reached, a consensus on how to respond to these patients may aid PCPs in their counseling.

### Limitations

A possible limitation is the response rate, especially among the researchers. Although these percentages are low, they are in line with those reported in similar Delphi studies or unsolicited questionnaires [52]. We included international researchers to realize a large and varied sample of expertise in order to obtain a large spectrum of responses. One may argue that this could lower the generalizability to the Dutch context; yet, as the effectiveness of the SCIs is very similar across countries, and international data are often used in the Netherlands in communication on the effectiveness of SCIs, we believe that the level of distortion caused by this choice is probably very low.

We tried to include both GPs and PNs in the PCP group, but the first round only PNs were included. All GPs from our first round declined participation by stating that they themselves did not provide much SCC and referred us to their practice’s PNs [7, 53]. By recruiting PCPs via the DRQSCP [25], we successfully managed to include a more diverse range of PCPs in our second and third rounds. Including a wide variety of participants is a strength of the study.

It may be possible that selectivity has occurred within the group of PNs, as participants who might have a particular interest in, or a strong opinion on this topic are more likely to participate. However, when inviting PCPs we tried to include a balanced mix of occupations of PCPs who provided active SCC, to ensure a heterogenic group. This may strengthen generalizability, mainly for the national setting, but perhaps also for a part for other countries, as primary care guidelines often are similar (for example the ‘5 As’ [10]) even if the setting or the execution may slightly differ (e.g. SCC not being provided by specially trained PNs but by more general educated GPs).

## Conclusions

This systematic exploration and consensus study focused on obtaining an overview of the knowledge and viewpoints on the effectiveness and use of SCIs different smoking cessation experts. The four objectives of this study were to gain an overview of (i) the criteria important for recommending an SCI, (ii) the perceptions on the effectiveness of SCIs, (iii) the criteria important for counseling different (high-risk) groups of smokers and (iv) the perceptions on the use of e-cigarettes as a means to quit. Based on a three-round Delphi-study, we found a high agreement among researchers and PCPs on which patient characteristic should be taken into account when choosing a fitting SCI for individual patients (e.g. taking into account the patient’s needs and previous cessation attempts). We also found that PCPs display a lower degree of consensus on the effectiveness of SCIs. Both researchers and PCPs see value in the use of special protocols for high-risk groups of patients, but the two groups did not reach consensus on the use of e-cigarettes as a means to quit. Making an inventory of PCPs’ needs regarding SCIs and their usage may provide insight into how to facilitate a better uptake in the primary care setting.

## Supplementary Material

cyab022_Supp

## Data Availability

The datasets used and/or analyzed during the current staq15udy are available from the corresponding author on reasonable request.

## List of abbreviations

SCC: Smoking cessation counseling; SCI: Smoking cessation intervention; PCP: Primary care professionals; GP: General practitioner; PN: Practice nurse; Mdn: Median; IQR: Interquartile range; DRQSCP: Dutch Register for Qualified Smoking Cessation Professionals

## Trial registration

Evaluation by the Medical Ethics Committee azM/UM revealed that no medical ethical clearance was needed according to the rules of the Medical Research Involving Human Subjects Act (WMO – 2017-0168).
