# Supplementary material for: Do professional perspectives on evidence-based smoking cessation methods align? A Delphi study among researchers and healthcare professionals
Source: Health Educ Res. 2022 Jan 22;36(4):434–45. doi: 10.1093/her/cyab022 (PMC8783547; doi:10.1093/her/cyab022)
Supplement: cyab022_Supp [file cyab022_supp.zip › cyab022_supp.docx]

| Appendix **Table II** Results per item of the second- and third-round of ‘research’ and ‘health professionals’ | | | | | | | | | | | | | | | | | | | | |
| --- | --- | --- | --- | --- | --- | --- | --- | --- | --- | --- | --- | --- | --- | --- | --- | --- | --- | --- | --- | --- |
|  |  | Research experts | | | | | | | | |  | PCPs | | | | | | | | |
|  |  | Second round | | | |  | Third round | | | |  | Second round | | | |  | Third round | | | |
|  |  | N | Mdn | IQR |  |  | N | Mdn | IQR |  |  | N | Mdn | IQR |  |  | N | Mdn | IQR |  |
| **Topic 1. Patient characteristics that should be taken into account when recommending an SCI (patient characteristics)** |  |  |  |  |  |  |  |  |  |  |  |  |  |  |  |  |  |  |  |  |
| The smoker’s preference for a specific (type of) intervention |  | 27 | 6.5 | **1** |  |  |  |  |  |  |  | 51 | 6.0 | **1** |  |  |  |  |  |  |
| The extent to which the characteristics of an intervention meet the smoker’s needs |  | 27 | 6.0 | **1** |  |  |  |  |  |  |  | 51 | 6.0 | **1** |  |  |  |  |  |  |
| The smoker’s background (such as educational level or health literacy skills) |  | 27 | 5.5 | **1** |  |  |  |  |  |  |  | 51 | 5.0 | **1** |  |  |  |  |  |  |
| The smoker’s personal characteristics (such as age, gender or overall lifestyle) |  | 27 | 5.5 | **1** |  |  |  |  |  |  |  | 51 | 5.5 | **1** |  |  |  |  |  |  |
| The (type of) intervention(s) the smoker has previously used |  | 27 | 6.0 | **1** |  |  |  |  |  |  |  | 51 | 6.0 | **1** |  |  |  |  |  |  |
| The number of quit attempts that the smoker has previously undertaken |  | 27 | 5.5 | **1** |  |  |  |  |  |  |  | 51 | 5.5 | **1** |  |  |  |  |  |  |
| The level of success of the smoker’s previous cessation attempts |  | 27 | 5.5 | **1** |  |  |  |  |  |  |  | 51 | 5.5 | **1** |  |  |  |  |  |  |
| The time elapsed since the smoker’s last smoking cessation attempt |  | 27 | 5.0 | **1** |  |  |  |  |  |  |  | 51 | 5.0 | 2 |  |  | 32 | 5.5 | **1** |  |
| The difficulties the smoker experienced in previous cessation attempts |  | 27 | 6.0 | **1** |  |  |  |  |  |  |  | 51 | 6.5 | **1** |  |  |  |  |  |  |
| The smoker’s motivation to quit smoking |  | 27 | 6.0 | **1** |  |  |  |  |  |  |  | 51 | 6.5 | **1** |  |  |  |  |  |  |
| The smoker’s level of nicotine addiction |  | 27 | 6.0 | **1** |  |  |  |  |  |  |  | 51 | 5.5 | **1** |  |  |  |  |  |  |
| The financial costs of using the intervention |  | 27 | 5.5 | **1** |  |  |  |  |  |  |  | 51 | 5.0 | 2 |  |  | 32 | 5.0 | **1** |  |
| **Topic 2. Criteria that should be met by an SCI when recommending an SCI for an individual patient (intervention characteristics)** |  |  |  |  |  |  |  |  |  |  |  |  |  |  |  |  |  |  |  |  |
| When the smoker is highly motivated to quit |  | 27 | 6.5 | **1** |  |  |  |  |  |  |  | 50 | 6.5 | **1** |  |  |  |  |  |  |
| When the intervention meets the smoker’s needs as perceived by the healthcare professional |  | 27 | 5.0 | **1** |  |  |  |  |  |  |  | 50 | 6.0 | **1** |  |  |  |  |  |  |
| When the intervention matches the smoker’s preferences |  | 27 | 6.0 | **1** |  |  |  |  |  |  |  | 50 | 6.0 | **0** |  |  |  |  |  |  |
| When the intervention is tailored to that what the smoker already knows about smoking cessation |  | 27 | 5.0 | 2 |  |  | 19 | 5.5 | **0** |  |  | 50 | 5.5 | **1** |  |  |  |  |  |  |
| When the intervention supports the smoker in developing self-control regarding smoking and smoking cessation |  | 27 | 6.0 | **1** |  |  |  |  |  |  |  | 50 | 6.0 | **0** |  |  |  |  |  |  |
| When the intervention includes more rather than fewer sessions. |  | 27 | 4.0 | 3 |  |  | 19 | 4.5 | **1** |  |  | 50 | 4.0 | **1** |  |  |  |  |  |  |
| When the intervention continues over a longer period of time |  | 27 | 5.0 | 2 |  |  | 19 | 5.0 | **1** |  |  | 50 | 5.0 | 2 |  |  | 32 | 4.5 | 2 |  |
| When the intervention includes highly detailed information on smoking cessation |  | 27 | 4.0 | 2 |  |  | 19 | 4.5 | 2 |  |  | 50 | 4.5 | **1** |  |  |  |  |  |  |
| When the intervention is used by the smoker as it is meant to be (intervention fidelity) |  | 27 | 5.5 | 2 |  |  | 19 | 6.0 | **1** |  |  | 50 | 5.5 | **1** |  |  |  |  |  |  |
| When an independent RCT study shows that the intervention significantly increases the likelihood of smoking cessation over 6 months or longer when compared to usual care |  | 27 | 6.0 | 2 |  |  | 19 | 6.0 | **0** |  |  | 49 | 5.5 | **1** |  |  |  |  |  |  |
| When an independent RCT study shows that the intervention significantly increases the number of quit attempts when compared to usual care |  | 27 | 5.0 | 2 |  |  | 19 | 5.5 | **1** |  |  | 49 | 5.0 | **1** |  |  |  |  |  |  |
| When the intervention is certified as being effective (for example by national smoking cessation associations) |  | 27 | 6.0 | **1** |  |  |  |  |  |  |  | 49 | 5.0 | 2 |  |  | 32 | 5.5 | **1** |  |
| When the intervention is recommended by national guidelines for tobacco cessation |  | 27 | 6.0 | **1** |  |  |  |  |  |  |  | 49 | 5.5 | **1** |  |  |  |  |  |  |
| **Topic 3. How is the effectiveness of existing SCIs perceived?** |  |  |  |  |  |  |  |  |  |  |  |  |  |  |  |  |  |  |  |  |
| Pharmacotherapy |  | 26 | 6.0 | 2 |  |  | 19 | 6.0 | **1** |  |  | 49 | 6.0 | 2 |  |  | 32 | 6.0 | 2 |  |
| E-cigarettes |  | 26 | 3.0 | 2 |  |  | 19 | 3.0 | 2 |  |  | 49 | 3.0 | **1** |  |  |  |  |  |  |
| Freely available nicotine replacement therapy (for example patches or chewing gum) |  | 26 | 6.0 | 2 |  |  | 19 | 6.0 | **1** |  |  | 49 | 5.5 | **1** |  |  |  |  |  |  |
| Acupuncture |  | 26 | 2.5 | 2 |  |  | 19 | 2.0 | **1** |  |  | 49 | 4.0 | 2 |  |  | 32 | 5.5 | 2 |  |
| Laser therapy |  | 26 | 2.5 | 2 |  |  | 19 | 1.5 | **1** |  |  | 49 | 3.5 | **1** |  |  |  |  |  |  |
| Brief cessation advice by a healthcare professional without additional interventions |  | 26 | 6.0 | 2 |  |  | 19 | 6.5 | **1** |  |  | 49 | 6.0 | 2 |  |  | 32 | 6.0 | **1** |  |
| Brief cessation advice by a healthcare professional in combination with pharmacotherapy |  | 26 | 5.0 | 2 |  |  | 19 | 5.0 | **0** |  |  | 49 | 5.0 | 2 |  |  | 32 | 5.5 | 2 |  |
| Behavioral counseling – face-to-face |  | 26 | 6.0 | 2 |  |  | 19 | 6.0 | 2 |  |  | 49 | 5.0 | 2 |  |  | 32 | 5.5 | 2 |  |
| Behavioral counseling – via telephone |  | 26 | 5.5 | **1** |  |  |  |  |  |  |  | 49 | 5.5 | **1** |  |  |  |  |  |  |
| Behavioral counseling – in groups |  | 26 | 6.0 | **1** |  |  |  |  |  |  |  | 49 | 5.5 | **1** |  |  |  |  |  |  |
| Behavioral counseling – eHealth |  | 26 | 5.5 | **1** |  |  |  |  |  |  |  | 49 | 5.0 | **1** |  |  |  |  |  |  |
| Relaxation exercises (for example mindfulness or yoga) |  | 26 | 4.5 | **1** |  |  |  |  |  |  |  | 49 | 4.5 | **1** |  |  |  |  |  |  |
| Quitting without any form of counseling or other resources |  | 26 | 3.0 | 3 |  |  | 19 | 3.0 | 2 |  |  | 49 | 4.0 | 2 |  |  | 32 | 4.5 | 3 |  |
| **Topic 4. Factors that should be taken into account when counseling different (high-risk) groups of smoking patients** |  |  |  |  |  |  |  |  |  |  |  |  |  |  |  |  |  |  |  |  |
| **Smokers with smoking related complaints or conditions** |  |  |  |  |  |  |  |  |  |  |  |  |  |  |  |  |  |  |  |  |
| The smoker should be informed about his or her health risk |  | 25 | 6.5 | 2 |  |  | 19 | 6.5 | **1** |  |  | 48 | 6.5 | **1** |  |  |  |  |  |  |
| The smoker should be informed about the health risks for others in their surroundings |  | 25 | 6.0 | **1** |  |  |  |  |  |  |  | 48 | 6.0 | **0** |  |  |  |  |  |  |
| When a smoker is motivated to quit, treatment should start as soon as possible |  | 25 | 7.0 | **0** |  |  |  |  |  |  |  | 48 | 6.0 | **1** |  |  |  |  |  |  |
| Counseling should be based on motivational interviewing techniques |  | 25 | 5.5 | 2 |  |  | 19 | 6.5 | **1** |  |  | 48 | 6.5 | **1** |  |  |  |  |  |  |
| Counseling should be tailored to the smoker’s individual health problems |  | 25 | 6.0 | **1** |  |  |  |  |  |  |  | 48 | 6.5 | **1** |  |  |  |  |  |  |
| This group of smokers should receive the same cessation support as smokers without complaints or conditions |  | 25 | 4.0 | 3 |  |  | 19 | 5.0 | 2 |  |  | 48 | 4.5 | **1** |  |  |  |  |  |  |
| **Smoking pregnant women** |  |  |  |  |  |  |  |  |  |  |  |  |  |  |  |  |  |  |  |  |
| The smoker should be informed about her health risk as well as the risks for the unborn child |  | 25 | 7.0 | **0** |  |  |  |  |  |  |  | 48 | 6.5 | **1** |  |  |  |  |  |  |
| When a smoker is motivated to quit, treatment should start as soon as possible |  | 25 | 7.0 | **0** |  |  |  |  |  |  |  | 48 | 6.5 | **1** |  |  |  |  |  |  |
| The smoker’s partner should be encouraged to provide cessation support |  | 25 | 6.5 | **0** |  |  |  |  |  |  |  | 48 | 6.5 | **1** |  |  |  |  |  |  |
| The smoker’s partner should also be encouraged to quit smoking |  | 25 | 7.0 | **0** |  |  |  |  |  |  |  | 48 | 6.5 | **1** |  |  |  |  |  |  |
| The smoker should be informed of the risks of smoking during pregnancy |  | 25 | 7.0 | **0** |  |  |  |  |  |  |  | 48 | 6.5 | **1** |  |  |  |  |  |  |
| This group of smokers should receive the same cessation support as non-pregnant smokers |  | 25 | 4.0 | 3 |  |  | 19 | 3.5 | 3 |  |  | 48 | 4.0 | 2 |  |  | 32 | 3.5 | 3 |  |
| **Smokers with a low SES** |  |  |  |  |  |  |  |  |  |  |  |  |  |  |  |  |  |  |  |  |
| The smoker should be informed about his or her health risk |  | 25 | 6.5 | **1** |  |  |  |  |  |  |  | 48 | 6.5 | **1** |  |  |  |  |  |  |
| The smoker should be informed of the money they could save by quitting |  | 25 | 6.0 | **1** |  |  |  |  |  |  |  | 48 | 6.0 | 2 |  |  | 32 | 6.0 | **1** |  |
| Support should be focused on planning and performing alternative behaviors (coping planning) |  | 25 | 6.0 | 2 |  |  | 19 | 6.0 | **1** |  |  | 48 | 6.0 | 2 |  |  | 32 | 6.0 | **0** |  |
| Counseling should be based on motivational interviewing techniques |  | 25 | 6.0 | 2 |  |  | 19 | 6.0 | 2 |  |  | 48 | 6.0 | **1** |  |  |  |  |  |  |
| This group of smokers should receive the same cessation support as smokers with an average SES |  | 25 | 4.0 | 3 |  |  | 19 | 4.5 | 3 |  |  | 48 | 4.5 | 2 |  |  | 32 | 4.5 | 3 |  |
| **Smokers with a low motivation to quit** |  |  |  |  |  |  |  |  |  |  |  |  |  |  |  |  |  |  |  |  |
| Counseling should focus first on increasing motivation (quitting should be attempted after motivation has increased) |  | 25 | 6.0 | **1** |  |  |  |  |  |  |  | 47 | 6.5 | **1** |  |  |  |  |  |  |
| Healthcare professionals should use motivational interview techniques |  | 25 | 6.0 | **1** |  |  |  |  |  |  |  | 47 | 6.5 | **1** |  |  |  |  |  |  |
| The smoker should be informed about his or her health risk |  | 25 | 6.5 | **1** |  |  |  |  |  |  |  | 47 | 6.0 | **1** |  |  |  |  |  |  |
| The smoker should be informed about the health risk for others in their environment |  | 25 | 6.0 | **1** |  |  |  |  |  |  |  | 47 | 6.0 | **1** |  |  |  |  |  |  |
| This group of smokers should receive the same cessation support as smokers who are highly motivated |  | 25 | 4.5 | 4 |  |  | 19 | 5.0 | 2 |  |  | 47 | 4.5 | 2 |  |  | 32 | 5.0 | 2 |  |
| **Topic 5. The use of e-cigarettes as a means to quit** |  |  |  |  |  |  |  |  |  |  |  |  |  |  |  |  |  |  |  |  |
| The healthcare provider should discourage using e-cigarettes as an aid for smoking cessation |  | 25 | 4.0 | 4 |  |  | 19 | 4.0 | 2 |  |  | 49 | 5.0 | 2 |  |  | 32 | 5.0 | 2 |  |
| The healthcare provider should inform the smoker fully about the use and risks of e-cigarettes before talking about them as a form of smoking cessation intervention |  | 25 | 6.0 | **1** |  |  |  |  |  |  |  | 49 | 5.0 | 2 |  |  | 32 | 5.5 | 2 |  |
| The caregiver can recommend e-cigarettes as an aid for smoking cessation, but not as the most preferred option |  | 25 | 5.5 | 2 |  |  | 19 | 5.0 | 2 |  |  | 49 | 4.0 | 3 |  |  | 32 | 4.0 | 2 |  |

| **Table III** Consensus between the groups of research experts and healthcare professionals | | | | | | |
| --- | --- | --- | --- | --- | --- | --- |
|  |  | **N** | **Mdn research** | **Mdn PCP** | **Wilcoxon W** | **Sig.** |
| **Topic 1. Patient characteristics that should be taken into account when recommending an SCI (patient characteristics)** |  |  |  |  |  |  |
| The smoker’s preference for a specific (type of) intervention |  | 78 | 6.5 | 6.0 | 1918.0 | .251 |
| The extent to which the characteristics of an intervention meet the smoker’s needs |  | **78** | **6.0** | **6.0** | **1841.0** | **.041** |
| The smoker’s background (such as educational level or health literacy skills) |  | 78 | 5.5 | 5.0 | 1992.0 | .802 |
| The smoker’s personal characteristics (such as age, gender or overall lifestyle) |  | 78 | 5.5 | 5.5 | 1972.0 | .633 |
| The (type of) intervention(s) the smoker has previously used |  | **78** | **6.0** | **6.0** | **868.5** | **.018** |
| The number of quit attempts that the smoker has previously undertaken |  | 78 | 5.5 | 5.5 | 896.0 | .054 |
| The level of success of the smoker’s previous cessation attempts |  | 78 | 5.5 | 5.5 | 939.5 | .162 |
| The time elapsed since the smoker’s last smoking cessation attempt |  | 61 | 5.0 | 5.5 | 940.0 | .165 |
| The difficulties the smoker experienced in previous cessation attempts |  | 78 | 6.0 | 6.5 | 1995.5 | .824 |
| The smoker’s motivation to quit smoking |  | 78 | 6.0 | 6.5 | 2009.0 | .948 |
| The smoker’s level of nicotine addiction |  | **78** | **6.0** | **5.5** | **1791.0** | **.010** |
| The financial costs of using the intervention |  | **61** | **5.5** | **5.0** | **1802.0** | **.020** |
| **Topic 2. Criteria that should be met by an SCI when recommending an SCI for an individual patient (intervention characteristics)** |  |  |  |  |  |  |
| When the smoker is highly motivated to quit |  | 77 | 6.5 | 6.5 | 988.0 | .411 |
| When the intervention meets the smoker’s needs as perceived by the healthcare professional |  | **77** | **5.0** | **6.0** | **657.5** | **.000** |
| When the intervention matches the smoker’s preferences |  | 77 | 6.0 | 6.0 | 1911.0 | .646 |
| When the intervention is tailored to that what the smoker already knows about smoking cessation |  | 70 | 5.0 | 5.5 | 925.0 | .127 |
| When the intervention supports the smoker in developing self-control regarding smoking and smoking cessation |  | 77 | 6.0 | 6.0 | 1886.5 | .461 |
| When the intervention includes more rather than fewer sessions. |  | 54 | 4.5 | 4.5 | 1044.5 | .925 |
| When the intervention continues over a longer period of time |  | 70 | 5.0 | 4.5 | 986.5 | .459 |
| When the intervention includes highly detailed information on smoking cessation |  | 70 | 4.5 | 5.5 | 1945.5 | .958 |
| When the intervention is used by the smoker as it is meant to be (intervention fidelity) |  | 70 | 6.0 | 5.5 | 951.5 | .262 |
| When an independent RCT study shows that the intervention significantly increases the likelihood of smoking cessation over 6 months or longer when compared to usual care |  | **69** | **5.0** | **4.5** | **1728.0** | **.071** |
| When an independent RCT study shows that the intervention significantly increases the number of quit attempts when compared to usual care |  | 69 | 6.0 | 5.5 | 900.0 | .112 |
| When the intervention is certified as being effective (for example by national smoking cessation associations) |  | **61** | **6.0** | **5.5** | **1676.0** | **.017** |
| When the intervention is recommended by national guidelines for tobacco cessation |  | **76** | **6.5** | **5.5** | **1644.0** | **.005** |
| **Topic 3. How is the effectiveness of existing SCIs perceived?** |  |  |  |  |  |  |
| Pharmacotherapy |  | 54 | 6.0 | 6.0 | 1839.0 | .786 |
| E-cigarettes |  | 69 | 3.0 | 3.0 | 912.0 | .385 |
| Freely available nicotine replacement therapy (for example patches or chewing gum) |  | 69 | 6.0 | 5.5 | 1717.5 | .092 |
| Acupuncture |  | **54** | **2.0** | **5.5** | **589.0** | **.000** |
| Laser therapy |  | **54** | **1.5** | **3.5** | **580.0** | **.000** |
| Brief cessation advice by a healthcare professional without additional interventions |  | 54 | 6.5 | 6.0 | 1705.0 | .066 |
| Brief cessation advice by a healthcare professional in combination with pharmacotherapy |  | 54 | 5.0 | 5.5 | 1795.0 | .446 |
| Behavioral counseling – face-to-face |  | **69** | **6.0** | **5.5** | **1525.0** | **.000** |
| Behavioral counseling – via telephone |  | 75 | 5.5 | 5.5 | 1744.5 | .172 |
| Behavioral counseling – in groups |  | **75** | **6.0** | **5.5** | **1626.5** | **.006** |
| Behavioral counseling – eHealth |  | **60** | **5.5** | **5.0** | **1647.5** | **.012** |
| Relaxation exercises (for example mindfulness or yoga) |  | 75 | 4.5 | 4.5 | 1854.5 | .930 |
| Quitting without any form of counseling or other resources |  | **54** | **3.0** | **4.0** | **753.0** | **.007** |
| **Topic 4. Factors that should be taken into account when counseling different (high-risk) groups of smoking patients** |  |  |  |  |  |  |
| **Smokers with smoking related complaints or conditions** |  |  |  |  |  |  |
| The smoker should be informed about his or her health risk |  | 68 | 6.5 | 6.5 | 1659.5 | .135 |
| The smoker should be informed about the health risks for others in their surroundings |  | 73 | 6.0 | 6.0 | 1633.5 | .070 |
| When a smoker is motivated to quit, treatment should start as soon as possible |  | **73** | **7.0** | **6.0** | **1414.0** | **.000** |
| Counseling should be based on motivational interviewing techniques |  | **68** | **6.5** | **6.5** | **739.0** | **.020** |
| Counseling should be tailored to the smoker’s individual health problems |  | 73 | 6.0 | 6.5 | 907.0 | .818 |
| This group of smokers should receive the same cessation support as smokers without complaints or conditions |  | 68 | 5.0 | 4.5 | 872.0 | .516 |
| **Smoking pregnant women** |  |  |  |  |  |  |
| The smoker should be informed about her health risk as well as the risks for the unborn child |  | **73** | **7.0** | **6.5** | **1609.0** | **.015** |
| When a smoker is motivated to quit, treatment should start as soon as possible |  | **73** | **7.0** | **6.5** | **1597.0** | **.007** |
| The smoker’s partner should be encouraged to provide cessation support |  | **73** | **6.5** | **6.5** | **1625.0** | **.047** |
| The smoker’s partner should also be encouraged to quit smoking |  | **73** | **7.0** | **6.5** | **1589.5** | **.012** |
| The smoker should be informed of the risks of smoking during pregnancy |  | 73 | 7.0 | 6.5 | 1680.0 | .150 |
| This group of smokers should receive the same cessation support as non-pregnant smokers |  | 54 | 3.5 | 4.0 | 1737.0 | .643 |
| **Smokers with a low SES** |  |  |  |  |  |  |
| The smoker should be informed about his or her health risk |  | 73 | 6.5 | 7.0 | 1703.5 | .345 |
| The smoker should be informed of the money they could save by quitting |  | 59 | 6.0 | 6.0 | 1755.0 | .794 |
| Support should be focused on planning and performing alternative behaviors (coping planning) |  | 54 | 6.0 | 6.0 | 1775.5 | .995 |
| Counseling should be based on motivational interviewing techniques |  | 68 | 6.0 | 6.0 | 779.5 | .071 |
| This group of smokers should receive the same cessation support as smokers with an average SES |  | 54 | 4.5 | 4.0 | 896.5 | .733 |
| **Smokers with a low motivation to quit** |  |  |  |  |  |  |
| Counseling should focus first on increasing motivation (quitting should be attempted after motivation has increased) |  | 72 | 6.0 | 6.5 | 890.0 | .768 |
| Healthcare professionals should use motivational interview techniques |  | 72 | 6.0 | 6.0 | 839.5 | .346 |
| The smoker should be informed about his or her health risk |  | **72** | **6.5** | **6.0** | **1558.5** | **.043** |
| The smoker should be informed about the health risk for others in their environment |  | 72 | 6.0 | 6.0 | 1578.5 | .087 |
| This group of smokers should receive the same cessation support as smokers who are highly motivated |  | 54 | 5.0 | 4.5 | 1694.5 | .799 |
| **Topic 5. The use of e-cigarettes as a means to quit** |  |  |  |  |  |  |
| The healthcare provider should discourage using e-cigarettes as an aid for smoking cessation |  | **54** | **4.0** | **5.0** | **760.5** | **.039** |
| The healthcare provider should inform the smoker fully about the use and risks of e-cigarettes before talking about them as a form of smoking cessation intervention |  | **59** | **6.0** | **5.5** | **1573.5** | **.002** |
| The caregiver can recommend e-cigarettes as an aid for smoking cessation, but not as the most preferred option |  | 54 | 5.0 | 4.0 | 1704.5 | .121 |
